# Dual Tumor Suppressor and Tumor Promoter Action of Sirtuins in Determining Malignant Phenotype

**DOI:** 10.3389/fphar.2019.00038

**Published:** 2019-01-30

**Authors:** Vincenzo Carafa, Lucia Altucci, Angela Nebbioso

**Affiliations:** Dipartimento di Medicina di Precisione, Università degli Studi della Campania Luigi Vanvitelli, Naples, Italy

**Keywords:** Sirtuins, epigenetics, cancer, EMT, cancer therapy

## Abstract

Sirtuins (SIRTs), class III histone deacetylases, are differentially expressed in several human cancers, where they display both oncogenic and tumor-suppressive properties depending on cellular context and experimental conditions. SIRTs are involved in many important biological processes and play a critical role in cancer initiation, promotion, and progression. A growing body of evidence indicates the involvement of SIRTs in regulating three important tumor processes: epithelial-to-mesenchymal transition (EMT), invasion, and metastasis. Many SIRTs are responsible for cellular metabolic reprogramming and drug resistance by inactivating cell death pathways and promoting uncontrolled proliferation. In this review, we summarize current knowledge on the role of SIRTs in cancer and discuss their puzzling dual function as tumor suppressors and tumor promoters, important for the future development of novel tailored SIRT-based cancer therapies.

## Introduction

Cancer is a leading cause of death worldwide, accounting for 8.8 million deaths in 2015, as recently reported by the World Health Organization. It is becoming increasingly evident that epigenetic alterations due to defects in chromatin modifiers and remodelers contribute to carcinogenesis (Nebbioso et al., 2018). The biological capabilities acquired by cells during malignant transformation were identified and denoted as the six “hallmarks of cancer”: sustaining proliferative signaling, evading growth suppressors, resisting cell death, enabling replicative immortality, inducing angiogenesis, and activating invasion and metastasis (Hanahan and Weinberg, 2000, 2011). In cell invasion and metastasis, alterations in cell-cell adhesions and cell shape are early processes responsible for the acquisition of invasive capabilities by a malignant cell. Epithelial-to-mesenchymal transition (EMT) is a dynamic and reversible transdifferentiation process that transforms an epithelial cell into a mesenchymal cell, endowing it with the ability to invade, escape apoptosis, and disseminate (Lamouille et al., 2014).

Epithelial-to-mesenchymal transition is classified into three different types: (i) type 1, which generates mesenchymal cells that undergo further differentiation into epithelial cells, and has a role in embryogenesis and organogenesis; (ii) type 2, which is involved in tissue repair after trauma and injury, and normally generates fibroblasts; (iii) type 3, which occurs in cancer progression and metastasis. EMT is activated by a number of transcriptional factors (TFs) and epigenetic regulators, including Sirtuins (SIRTs), members of the class III histone deacetylase family.

Several studies describe a correlation between cellular glucose metabolism and tumorigenesis, as in order to sustain energetic demands due to increased cell proliferation, cancer cells need to readjust their cellular metabolism (Sebastian and Mostoslavsky, 2015). Reprogramming of energy metabolism was recently introduced into the list of cancer hallmarks, increasing the complexity of the disease (Hanahan and Weinberg, 2011). Adjustments of energy metabolism sustain the rapid and uncontrolled growth and proliferation of cancer cells. While normal cells commonly switch from aerobic to anaerobic status by changing their glucose metabolism, cancer cells display elevated glycolysis and glutaminolysis, with an increase in and accumulation of glycolytic intermediates, as fuel for macromolecular synthesis leading to growth in tumor mass (Jeong and Haigis, 2015). Despite efforts to gain a greater insight into how this reprogramming can affect cancer development, its function remains unclear and controversial (Liberti and Locasale, 2016). Emerging evidence highlights a crucial role for SIRTs in this process (Bosch-Presegue and Vaquero, 2011; Chalkiadaki and Guarente, 2015; O’Callaghan and Vassilopoulos, 2017). Growing awareness of the pivotal role of epigenetic alterations in initiation, promotion, and progression of human cancers has led to a better understanding of the role of these epi-enzymes in driving human disease. Here, we summarize recent advances in our knowledge of the role of SIRTs in carcinogenesis. We also discuss their dual role as tumor promoters and tumor suppressors in cancer, including their involvement in EMT and energy metabolism programs.

## Sirtuin Family

Mammalian SIRTs include seven proteins (SIRT1-7) with deacetylase activity belonging to the class III histone deacetylase family. SIRTs share homology with the yeast deacetylase Sir2, and have different sequences and lengths in both their N- and C-terminal domains (Carafa et al., 2012). Expressed from bacteria to humans (Vaquero, 2009), SIRTs target histone and non-histone proteins. Although their best-characterized enzymatic activity is NAD^+^-dependent lysine deacetylation, SIRTs also catalyze other reactions, below discussed (Table 1).

**Table 1:** Classification of SIRTs including localization, enzymatic activity, targets, and involvement in cancer.

Sirtuin	Principal cellular localization	Biochemical activity	Histone targets	Protein targets	Association with cancer	
					Up-regulation	Down-regulation
SIRT1	Nucleus	Deacetylase	H1-K26Ac	FOXO family members, KU70, P53, GATA4-5, MYC, HIF-1α, MLH1, RB, P73, STAT3	Prostate, breast, lung, colon, ovarian, gastric, lymphoblastic leukemia, chronic lymphocytic leukemia, diffuse large B-cell leukemia, chronic myeloid leukemia, melanoma	Glioma, bladder, prostate, ovarian, triple negative breast cancer
			H3-K9Ac			
			H4-K16Ac			
			H4-K20me			
			H3-K9me3			
SIRT2	Cytoplasm	Deacetylase	H3-K18Ac	MYC, HIF-1α, α-tubulin, KRAS, CDH, CDC20, PEPCK, FOXO1, FOXO3, P53	Leukemia, neuroblastoma, hepatocellular carcinoma pancreatic, breast	Glioma, neck squamous cell carcinoma, breast, prostate, esophageal, ovarian, breast melanoma
			H3-K56Ac			
			H4-K16Ac			
SIRT3	Mitochondria	Deacetylase		SOD2, IDH2, FOXO3a, KU70, P53, HIF-1α	Colon, gastric, oral squamous, esophageal, renal, bladder, melanoma	Colon, breast, gastric, leukemia
SIRT4	Mitochondria	ADP-ribosylase		E-cadherin, MLYCD	Colorectal, Burkitt lymphoma	Bladder, breast, colon stomach, ovarian, thyroid
SIRT5	Mitochondria	Deacetylase desuccinylase deglutarylase demalonylase		CSP1 SOD1	Non-small cell lung, colorectal	Lung
SIRT6	Nucleus	Deacetylase ADP-ribosylase deacylase demyristoylase depalmitoylase	H3-K9Ac	PARP1, FOXO3a, NRF1, MYC, HIF-1α	Lung, prostate, melanoma, non-melanoma skin cancer	Pancreatic, breast, colon, hepatocellular carcinoma
			H3-K56Ac			
			H3-K18Ac			
SIRT7	Nucleus	Deacetylase desuccinylase	H3-K18Ac	HIF-1α, P53	Ovarian, colorectal, thyroid, prostate, breast, osteosarcoma, hepatocellular carcinoma	

Localization of SIRTs is restricted to three different subcellular compartments: cytoplasm, nucleus, and mitochondria. SIRT1, SIRT6, and SIRT7 are principally localized in the nucleus, SIRT2 in the cytosol, and SIRT3, SIRT4, and SIRT5 in the mitochondria. Different studies report the ability of some SIRTs to re-localize under different conditions (e.g., cell cycle phase, tissue type, developmental stage, stress condition, and metabolic status), suggesting the important role of their localization in regulating specific pathways (McGuinness et al., 2011). Specifically, SIRT1, SIRT2, and SIRT7 are often found in both the nucleus and cytoplasm (Michishita et al., 2005).

**FIGURE 1:**
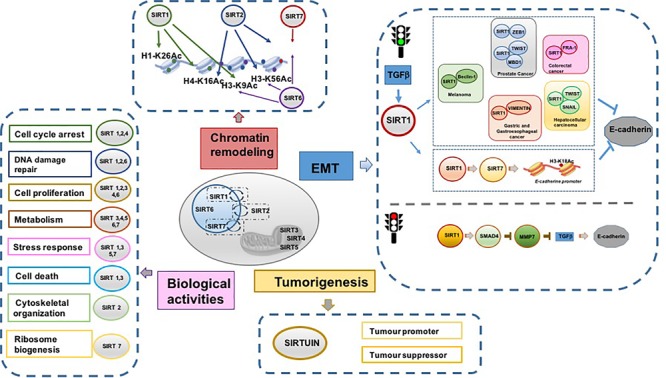
Schematic representation of principal SIRT functions.

Sirtuins modulate different pivotal cellular pathways such as DNA repair, transcriptional regulation, metabolism, aging, and senescence. As these biological processes are involved in cancer initiation and progression, interest in SIRTs as targets in cancer research has increased. Interestingly, in terms of energy metabolic reprogramming, emerging evidence shows the complex association of two metabolism-associated TFs, MYC and hypoxia inducible factor-1 (HIF-1), with some SIRTs (Zwaans and Lombard, 2014; Figure 1).

It is well documented that SIRTs act as tumor suppressors or tumor promoters (oncogenes) by modulating cell proliferation, differentiation, and death. Their different biological function in cancer depends on cell context and experimental conditions. Although the dual role of SIRTs is crucial in cancer biology, it remains a highly debated and controversial topic. Whether SIRTs act as tumor suppressors or promoters depends on (i) their different expression levels in tumors; (ii) their effect on cell cycle, cell growth, and cell death; (iii) their action on specific proto-oncogene and oncosuppressor proteins (Deng, 2009).

## Sirtuin Reactions

The most known and well-studied enzymatic reaction catalyzed by SIRTs is NAD^+^-dependent deacetylation, but others have been reported (Table 1). Deacetylation reaction begins with amide cleavage from NAD^+^ with the formation of nicotinamide and an intermediate of reaction, O-ADP-ribose. This intermediate is necessary for the deacetylation process by which SIRTs catalyze the transfer of one acetyl group from a lysine to O-ADP-ribose moiety to form *O*-acetyl-ADP-ribose and the deacetylated lysine product. This reaction consumes a mole equivalent of NAD^+^ per acetyl group removed and is controlled by the cellular [NAD]/[NADH] ratio (Sauve, 2010; Shi et al., 2013).

Although SIRT enzymes are known primarily as protein deacetylases, among the seven mammalian SIRTs only SIRT1, SIRT2, and SIRT3 possess a robust deacetylase activity. Other SIRTs (SIRT4, SIRT5, SIRT6, and SIRT7) exhibit a weak or no detectable deacetylation activity.

SIRT4 and SIRT6 display another well-described and well-studied enzymatic reaction, ADP-ribosyltransferase activity, by which they transfer a single ADP-ribosyl group from NAD^+^ to proteins (Sauve, 2010). Mechanistically, ADP-ribosylation and deacetylation reactions are similar because they cleave NAD^+^, thereby releasing nicotinamide. By ADP-ribosylation, SIRT4 and SIRT6 regulate the activity of glutamate dehydrogenase and PARP, respectively (Haigis et al., 2006; Pan et al., 2011).

It was recently reported that some SIRTs are able to catalyze other enzymatic reactions by removing different acyl groups. The first SIRT found to have a novel enzymatic activity was SIRT5, which exhibits weak physiological deacetylation but efficient demalonylation and desuccinylation activities. SIRT5 preferentially deacylates negatively charged carboxylate acyl groups by removing carboxylacyl-lysine. Studies performed on the crystal structure of SIRT5 confirmed that it is an NAD-dependent demalonylase and desuccinylase (Du et al., 2011). Its demalonylase or desuccinylase activity is much greater than its deacetylase activity. The best known acyl-CoA molecules with a carboxylate group are malonyl-CoA, made from acetyl-CoA by acetyl-CoA carboxylase and succinyl-CoA, an intermediate of the Krebs cycle. The preference for negatively charged acyl groups can be explained by the presence of two amino acid residues, Tyr102 and Arg105, in the active site, which are well conserved in most SIRTs (Yang et al., 2015). Another activity described for SIRT5 is glutarylation, which involves the removal of glutaryl-CoA, a metabolite of amino acid metabolism, structurally similar to succinyl-CoA and malonyl-CoA (Tan et al., 2014).

The discovery of these novel enzymatic properties of this class of proteins suggested that all SIRTs with a weak deacetylase activity may preferentially act on other acyl lysine modifications (Carafa et al., 2016). This observation led to the discovery of the defatty-acylation activity of SIRT6. Similar to PfSir2A, the *Plasmodium falciparum* SIRT, SIRT6 is able to efficiently hydrolyze different long-chain fatty acyl groups such as acetyl, malonyl, succinyl, butyryl, myristoyl, and palmitoyl several hundred-fold more efficiently than hydrolyzing acetyl groups. Through these reactions, SIRTs are able to regulate several key cellular processes (Jiang et al., 2013; Zhang et al., 2017b).

## Role of Sirtuins in EMT

The principal property of invasive cancer is tumor metastasis, resulting from the activation of EMT. This transdifferentiation process changes a polarized epithelial cell into a mesenchymal cell, which is able to migrate away from the epithelium in which it originated due to its increased migratory and invasive capacities. EMT is a reversible process caused by widespread epigenetic reprogramming of gene expression. Its counterpart is mesenchymal-to-epithelial transition (MET; Lamouille et al., 2014). Regardless of differences in tissue and signaling context, EMT is activated by EMT-TFs and epigenetic regulators controlling expression of proteins involved in cell polarity, cell–cell adhesion, cytoskeleton architecture, and extracellular matrix degradation. In addition, the maintenance of a stable mesenchymal phenotype depends on the level of histone acetylation and DNA methylation regulating the interconversion of heterochromatin into euchromatin, and vice versa. A growing body of evidence points to SIRTs as key epigenetic modulators of EMT activation and maintenance. However, SIRTs also display a contradictory role in EMT regulation, either promoting or suppressing this process (Palmirotta et al., 2016; O’Callaghan and Vassilopoulos, 2017; Sun et al., 2018), although the activation or repression of different cellular pathways in which they are involved depend on cellular context, stage of cancer, tissue of origin, and microenvironment. The best-characterized event occurring in EMT is loss of the fundamental cell–cell adhesion protein E-cadherin. E-cadherin loss correlates with poor prognosis, lower survival, and high rate of metastasis (Sun et al., 2018).

The positive regulation of EMT is mediated by TGF-β. TGF-β upregulates SIRT1, which in turn determines downregulation or degradation of E-cadherin by interacting with other TFs, promoting resistance to cell death, and cancer cell migration and invasion (Palmirotta et al., 2016). Several recent studies demonstrated that SIRT1 is involved in EMT activation by inactivating E-cadherin expression. miR-217 and SIRT1 play a key role in regulating EMT in chronic pancreatitis and pancreatic cancer (Deng et al., 2014). In particular, TGF-β1 was shown to induce EMT by downregulating miR-217 and upregulating SIRT1, leading to degradation of E-cadherin. miR-217 was shown to function as a tumor suppressor in pancreatic ductal adenocarcinoma by targeting KRAS (Zhao et al., 2010), and is downregulated and associated with poor survival in clear cell renal cell carcinoma (Li et al., 2013) and in gastric cancer (Chen et al., 2015).

SIRT1 regulates EMT in prostate cancer cells by interacting with the EMT-TF ZEB1 (Byles et al., 2012). Specifically, ZEB1 recruits SIRT1 to the *E-cadherin* promoter, leading to its gene suppression by deacetylating histone H3 and decreasing RNA polII binding. In addition, methyl-CpG binding domain protein 1 (MBD1) has an important function in pancreatic cancer, where it is upregulated and correlates with lymph node metastasis and poor survival (Xu et al., 2013). Mechanistically, MBD1 is associated with TWIST and SIRT1, via the TWIST-MBD1-SIRT1 complex on E-cadherin, which results in reduced E-cadherin transcription activity and induction of EMT. Significantly, targeting MBD1 reverses the EMT phenotype of pancreatic cancer and restores sensitivity to chemotherapy. SIRT1 is upregulated in the majority of hepatocellular carcinomas (HCCs) and enhances the invasive and metastatic potential of HCC by activating EMT markers, such as SNAIL, TWIST, and VIMENTIN, and inhibiting E-cadherin. SIRT1 expression is correlated with an unfavorable prognosis in patients with HCC (Hao et al., 2014; Serrano-Gomez et al., 2016), suggesting a potential therapeutic use for selective SIRT1-targeting drugs.

In gastric cancer metastasis, SIRT1-mediated downregulation of miR-204 inactivates LKB1, promoting cell invasion. Overexpression of miR-204 and knock-down of SIRT1 induce an MET phenotype by increasing E-cadherin and decreasing VIMENTIN levels, and inhibit gastric cancer metastasis (Zhang et al., 2013). The promotion of gastric cancer cell metastasis mediated by miR-204 downregulation suggests that this miR acts as a tumor suppressor and may represent a potential target in gastric cancer therapy (Zhang et al., 2013; Shrestha et al., 2017). Additional studies suggest miR-204 as useful target in treatment of glioblastoma (Song et al., 2016) and breast cancer (Shen et al., 2017).

In melanoma, SIRT1 was reported to promote EMT by downregulation of E-cadherin and its degradation via autophagy, due to deacetylation of BECLIN-1 (Sun et al., 2018). In colorectal cancer, EMT is activated by SIRT1 and the EMT-TF FRA-1 (Cheng et al., 2016).

In 2015, SIRT7 was identified as an important regulator of metastasis. SIRT7 is recruited by SIRT1 on the *E-cadherin* promoter, triggering deacetylation of histone H3-K18Ac with consequent transcriptional repression of downstream targets (Malik et al., 2015). The maintenance of malignant properties was also found to be a consequence of deacetylation of H3-K18Ac by SIRT7 (Barber et al., 2012). Consistent with these findings, SIRT7 overexpression promotes the development and progression of human colorectal cancer (Yu et al., 2014).

As tumor suppressors, SIRTs are also reported as negative modulators of EMT. SIRT1 overexpression in breast cancer cells reduces EMT in nude mice, while SIRT1 repression increases EMT (Simic et al., 2013). The repressive role of SIRT1 in EMT was also described in kidney tubular epithelial cells overexpressing SIRT1, which maintained the epithelial phenotype after TGF-β treatment (Simic et al., 2013). Conversely, silencing of SIRT1 increased EMT, as confirmed by E-cadherin decrease. Significantly, this function of SIRT1 was also observed in fibrosis following kidney injury. This effect was found mitigated in murine kidney tubular epithelial cells overexpressing SIRT1, and increased in SIRT1 knock-down cells. The absence of SIRT1 leads to hyperactivation of TGF-β and hyperacetylation of SMAD4, which in turn increases the expression of its target MMP7. Higher levels of MMP7 result in degradation of E-cadherin, thereby releasing β-catenin which then translocates to the nucleus, determining a mesenchymal phenotype (Simic et al., 2013).

The effect of SIRT1 on MMP7 expression via deacetylation of SMAD4 was also found in oral squamous cell carcinoma (OSCC) (Chen et al., 2014). The overexpression of SIRT1 or its activation by resveratrol, a SIRT activator, inhibits the migration, invasion, and metastasis of OSCC cells, with an increase in E-cadherin expression and decrease in mesenchymal markers. This repressive effect is due to the hypoacetylation of SMAD4 followed by inhibition of TGF-β signaling and MMP7 repression (Chen et al., 2014). Resveratrol was also found to play an inhibitory role in EMT in renal injury and fibrosis both *in vitro* and *in vivo*. Resveratrol-mediated SIRT1 upregulation attenuated renal injury and fibrosis by inhibiting TGF-β pathway via MMP7 through deacetylation of SMAD4 (Xiao et al., 2016).

Evidence of SIRT1 repressive action in EMT via inhibition of cell migration was also described in lung and ovarian cancer cells (Sun et al., 2013a,b). Specifically, SIRT1 activation by resveratrol hampers cancer metastasis *in vitro* and *in vivo* by blocking EMT.

Hypoxia inhibits SIRT1 expression by promoting its transcriptional repressor hypermethylated in cancer-1 (HIC1) binding on the SIRT1 proximal promoter in a SUMOylation-dependent manner, preventing binding with the transcriptional activator SP1. Disrupting SUMOylation by targeting either UBC9 or PIASy, the E2 and E3 small ubiquitin-like modifier (SUMO)-conjugating enzymes restored SIRT1 expression and promoted an epithelial-like phenotype of cancer cells, thereby arresting metastasis (Sun et al., 2013b). Decreased SIRT1 levels combined with elevated PIASy expression is implicated in more invasive types of cancers. SIRT1 suppresses hypoxia-induced EMT in nasal polyp formation (Lee et al., 2016). This repressive role is due to deacetylation of hypoxia-inducible factor 1 α (HIF-1α). Tissue-specific SIRT1 knock-down restores polyp formation in transgenic mice.

Other SIRTs are known to play a role in EMT. In non-malignant cells, SIRT2 was found to inhibit WNT signaling by direct binding to β-catenin. This interaction increases upon oxidative stress induced by ionizing radiation, inhibiting the expression of WNT target genes. An increase in MMP9 and a decrease in E-cadherin promoting cellular migration and invasion were observed in SIRT2-null cells (Nguyen et al., 2014).

The repressive role of SIRT3 and SIRT4 in EMT is associated with their ability to reprogram the energy metabolism. Specifically, it was demonstrated that the reciprocal interplay between cancer-associated fibroblasts (CAFs) and prostate cancer cells results in a mutual metabolic reprogramming (Fiaschi et al., 2012). Upon contact, both cell types undergo metabolic reprogramming, with CAFs and prostate cancer cells shifting toward a more glycolic and more aerobic metabolism, respectively. This process is controlled by HIF-1α, which drives redox- and SIRT3-dependent stabilization of HIF-1α in normoxic conditions. Lactate extruded from CAFs shuttles back to prostate cancer cells, which gradually became independent of glucose consumption while developing dependence on lactate upload to drive anabolic pathways and therefore cell growth. SIRT4 overexpression was shown to block proliferation, migration, and invasion of colon cancer cells via inhibition of glutamine metabolism (Miyo et al., 2015). Specifically, repression of glutamine metabolism drives the upregulation of E-cadherin expression and inhibits cell motility. Further, a decrease in SIRT4 expression is closely associated with the progression and recurrence of colorectal cancer.

SIRT7 overexpression inhibits EMT in OSCC, where SIRT7 expression correlates inversely with patient survival. Overexpression of SIRT7 strongly decreased OSCC migration and invasion, increased E-cadherin and downregulated VIMENTIN and MMP7 protein levels by deacetylating SMAD4, a key regulator of EMT (Levy and Hill, 2005; Ioannou et al., 2018; Li et al., 2018). In contrast with other studies (Barber et al., 2012; Yu et al., 2014; Malik et al., 2015), this report provides evidence of a new role for SIRT7 in tumor metastasis. In agreement, an association was found between SIRT7, breast cancer, and lung metastasis (Tang et al., 2017). Resveratrol treatment inhibits breast cancer and lung metastasis, and increases survival by activating SIRT7. A plausible explanation of this controversial function of SIRT7 in tumorigenesis could be derived from its dynamic regulation during tumor development, where high SIRT7 levels may initially contribute to oncogenic transformation and tumor growth (Aljada et al., 2015), while inhibit migration and invasion at later stages of cancer progression (Tang et al., 2017).

## Sirtuins and Cancer

Sirtuins are predominantly located in either nucleus (SIRT1, SIRT6, SIRT7), cytoplasm (SIRT2), or mitochondria (SIRT3, SIRT4, SIRT5).

### Nuclear Sirtuins

#### SIRT1

SIRT1 is the proto member of the SIRT family, and is the best known and most studied. SIRT1 is located primarily in the nucleus, is able to deacetylate histone and non-histone proteins, and is involved in several biological processes. SIRT1 regulates histone deacetylation and methylation through deacetylation of lysine 26 on histone H1 (H1-K26Ac), lysine 9 on histone H3 (H3-K9Ac), and lysine 16 on histone H4 (H4-K16Ac). It also deacetylates several non-histone proteins involved in cell cycle regulation, cell death induction, and metabolism, such as FOXO family members and KU70. In addition, SIRT1 affects histone methylation levels via production of mono-methylated histone H4 in lysine 20 (H4-K20me1) and tri-methylated histone H3 in lysine 9 (H3-K9me3) (Zhang and Kraus, 2010; Table 1).

Elevated expression of SIRT1 was observed in several cancer cell lines, and is generally associated with poor prognosis and overall survival (Wang et al., 2017). SIRT1 interacts with P53, triggering its deacetylation in Lys382 residue, and determines a block of all P53-dependent pathways, leading to uncontrolled cell cycle and inactivation of the apoptotic process (Vaziri et al., 2001). Chemo-resistance is a phenomenon observed in tumors overexpressing SIRT1, determining hyper proliferation and survival of cancer cells.

SIRT1 plays a dual role in tumorigenesis. An increasing number of studies report that SIRT1 has a function in metastasis and invasiveness in several cancers. The deacetylation of many proteins involved in tumor suppressor processes or DNA damage repair, and the inactivation of specific pathways support the role of SIRT1 as a tumor promoter. SIRT1 is involved in the initiation, promotion, and progression of several malignant tumors including prostate cancer (Jung-Hynes et al., 2009), breast cancer (Jin et al., 2018), lung cancer (Han et al., 2013), leukemia (Chen and Bhatia, 2013), colon cancer (Lin and Fang, 2013), melanoma (Ohanna et al., 2014), and ovarian and gastric cancer (Han et al., 2013; Shuang et al., 2015). *In vitro* experiments demonstrate that the inhibition of SIRT1 by treatment with small molecule SIRT1 inhibitors determines a significant decrease in cell growth, proliferation and viability (Wilking et al., 2014).

In gastric tumors, high expression levels of SIRT1 are associated with malignant status and poor survival. Studies of the molecular mechanisms responsible for gastric cancer progression highlight SIRT1 association with STAT3 (Nie et al., 2009; Zhang et al., 2017a). SIRT1 deacetylates STAT3, suppressing its inhibitory effect on gluconeogenesis. This interaction leads to an acceleration of the malignant process by activating proteins involved in cell survival, downregulating tumor suppressor genes, and conferring drug resistance.

SIRT1 expression is found increased in hematopoietic malignancies such as T-cell acute lymphoblastic leukemia, chronic lymphocytic leukemia, diffuse large B-cell leukemia, and chronic myeloid leukemia (CML) (Jang et al., 2008; Wang et al., 2011; Kozako et al., 2012). Specifically, several studies demonstrated the importance of the interaction between SIRT1 and onco-fusion proteins driving these pathologies. In CML, the onco-fusion protein BCR-ABL, generated by STAT5-mediated SIRT1 activation, plays a crucial role in malignant transformation and development of hematopoietic progenitor cells. SIRT1 promotes cell survival and cancer progression by deacetylating multiple substrates including FOXO1, P53, and KU70 (Yuan et al., 2012). Inhibition of SIRT1 might therefore selectively reduce survival and growth of CML stem cells and increase their responsiveness to clinical treatment with tyrosine kinase inhibitors. In acute myeloid leukemia, patients harboring the chromosomal translocation t(8;21)(q22;q22), AML1-ETO fusion protein binds the promoter region of *SIRT1* gene and is responsible for its activation and overexpression (Zhou et al., 2017).

SIRT1 also acts as a tumor suppressor via direct interaction with and consequent repression of other oncogenes, such as c-MYC (Yuan et al., 2009). In addition, a variety of human cancers including glioma, bladder, prostate, and ovarian cancer display decreased levels of SIRT1 (Wilking and Ahmad, 2015). Surprisingly, some studies report an opposing role for SIRT1 in the same tumor types. High protein and mRNA expression levels of SIRT1 in hormone receptor-positive and HER2 breast cancer subtypes demonstrate its oncogenic role (Rifai et al., 2017). In contrast, SIRT1 acts as a tumor suppressor in triple negative breast cancer cells, where it determines a block of cancer proliferation and cell growth (Yi et al., 2013). Conflicting findings were also reported in human prostate cancer cell lines, where SIRT1 pharmacological inhibition induces cell death, and reduces tumor growth and chemo-resistance (Long et al., 2014). Conversely, recent studies with SIRT1^-/-^ mice showed increased survival (Di Sante et al., 2015). This opposite behavior may be explained by the different role of SIRT1 in examined species (human vs murine), but requires further investigation.

#### SIRT6

SIRT6 shows low deacetylation and ADP-ribosylation activities. In addition, it was recently found to exhibit deacylase activity by removing myristoyl and palmitoyl groups (long-chain fatty acyl groups) from lysine residues (Feldman et al., 2013). SIRT6 is mainly located in the nucleus, where it binds and deacetylates chromatin, nucleosomes, and many TFs. It is also present in the endoplasmic reticulum, where it regulates tumor necrosis factor α by removing myristoyl groups from lysines 19 and 20, leading to its secretion from macrophages (Jiang et al., 2013). SIRT6 deacetylates lysine 9 on histone H3 (H3-K9Ac) and lysine 56 on histone H3 (H3-K56Ac) (Michishita et al., 2008; Michishita et al., 2009). Lysine 18 on histone H3 (H3-K18Ac) was recently identified as a new substrate of SIRT6 (Tasselli et al., 2016).

The principal function of SIRT6 is the control of cellular homeostasis by regulating DNA-damage repair, telomere maintenance, and metabolism. SIRT6 involvement in the DNA-damage repair pathway is principally due to its ADP-ribosyltransferase activity on poly-ADP-ribose-polymerase under stress conditions (Mao et al., 2011). The action of SIRT6 is strictly correlated to that of SIRT1. Upon nutritional stress, SIRT1 regulates SIRT6 by forming a complex with other proteins involved in metabolism regulation, such as FOXO3a and NRF1. Like SIRT1, SIRT6 is also able to reduce the transcription of MYC and HIF-1 in cancer cells by reprogramming glucose metabolism (Palmirotta et al., 2016).

Similarly, SIRT6 involvement in tumorigenesis is tissue-context specific, and displays both functions of tumor promoter and tumor suppressor (Qu et al., 2017). SIRT6 tumor suppressor activity is documented in pancreatic cancer (Kugel et al., 2016), breast cancer, colon cancer (Ioris et al., 2017), and HCCs (Zhang and Qin, 2014). In these tumors, expression levels of SIRT6 are decreased and aerobic glycolysis pathways are blocked. In other cancer types, such as lung cancer (Desantis et al., 2018), prostate cancer (Bai et al., 2016), melanoma, and non-melanoma skin cancer (Garcia-Peterson et al., 2017), SIRT6 is upregulated both at mRNA and protein level, and acts as an oncogene responsible for cancer cell proliferation.

#### SIRT7

SIRT7 is the last deacetylase discovered and is therefore the least well characterized. SIRT7 deacetylates lysine 18 on histone H3 (H3-K18Ac) as well as non-histone proteins. Recent studies reported that SIRT7 also exhibits desuccinylase activity, acting after a genomic insult on lysine 122 of histone H3 (Li et al., 2016). SIRT7 has a role in the control of ribosomal RNA expression (Zhang et al., 2016). It is localized in the nucleus, where it participates in the activation of RNA polI (Wei et al., 2017). SIRT7 is involved in seven cellular pathways regulating metabolism control, genome stability, aging, stress response, transcription, ribosome biogenesis, and tumorigenesis (Blank and Grummt, 2017). SIRT7 has an oncogenic role in tumorigenesis, activating cancer proliferation via deacetylation of specific promoters of different tumor suppressor genes. SIRT7 is a biomarker for aggressive and metastatic tumors with poor clinical outcome, and its overexpression correlates with advanced tumor stage. SIRT7 was found overexpressed and upregulated in several tumors, including ovarian cancer (Wang et al., 2015), colorectal cancer, osteosarcoma (Wei et al., 2017), prostate cancer (Haider et al., 2017), breast cancer (Geng et al., 2015), and HCC (Kim et al., 2013). SIRT7 knock-down determines a reduction in tumor invasiveness and progression, and an induction of apoptotic pathways.

The oncogenic properties of SIRT7 may be due to its interaction with P53, a secondary effect observed after upregulation of rRNA synthesis, determining rapid tumor growth (Ford et al., 2006; Blank and Grummt, 2017).

### Cytoplasmic Sirtuins

#### SIRT2

SIRT2 is mainly localized in the cytoplasm, but translocates rapidly to the nucleus during G2/M cell cycle transition. In the nucleus, SIRT2 binds chromatin and deacetylates histone and non-histone proteins, controlling cell cycle progression. SIRT2 deacetylates lysine 18 on histone H3 (H3-K18Ac), lysine 56 on histone H3 (H3-K56Ac) and lysine 16 on histone H4 (H4-K16Ac) (Table 1). The different cellular localization of SIRT2 is responsible for different biological downstream effects causing many pathologies including cancer. SIRT2 is involved in regulation of mitotic processes, cell motility, differentiation, oxidative metabolism, and cell death (Inoue et al., 2007a). It plays a fundamental role in cytoskeletal organization by deacetylating α-tubulin. In tumorigenesis, SIRT2 shows both tumor suppressor and oncogenic activities. Its tumor suppressor action is the result of the deacetylation of many proteins involved in important biological pathways such as cell proliferation, cell integrity, and DNA damage (Huang et al., 2017). In addition, SIRT2 acts as a checkpoint and prevents chromatin condensation and hyperploid cell formation through its ability to regulate mitotic integrity. In several cancer cell lines, including glioma (Inoue et al., 2007b), neck squamous cell carcinoma (Lai et al., 2013), non-small cell lung cancer (Grbesa et al., 2015), breast cancer (McGlynn et al., 2014), prostate cancer (Kim et al., 2011), and HCC (Chen et al., 2013), *SIRT2* gene is found downregulated or deleted. Recent evidence highlights the role of SIRT2 in serous ovarian carcinoma, where its reduced expression is responsible for cancer progression, promoting cell migration, invasion, lymph node metastasis, and peritoneal dissemination (Du et al., 2017). In breast cancer progression, SIRT2 function depends on the grade and classification of the tumor. Specifically, in moderately differentiated grade 2 breast tumors, SIRT2 expression is very low and G2/M phase transition is deregulated, correlating with poor prognosis. In this scenario, SIRT2 exerts a tumor suppressor function. Conversely, in poorly differentiated breast cancers, such as grade 3 breast tumors, SIRT2 expression levels are high and are responsible for further dysregulation of cell cycle progression and DNA repair processes. This correlates with a more aggressive tumor, shorter time to relapse and death, highlighting the oncogenic role of SIRT2 (McGlynn et al., 2014). Tumors with high levels of SIRT2 were found resistant and refractory to chemotherapy. In leukemia, neuroblastoma, HCC, and pancreatic cancer, *SIRT2* gene is upregulated and is responsible for vascular invasion, cell proliferation, and tumor growth. SIRT2 induces myeloid differentiation and deacetylates KRAS, increasing the tumorigenesis process, in association with nicotinamide phosphoribosyltransferase and HDAC6, respectively (Yang et al., 2013). A feedback interaction is also described between SIRT2 and N-MYC in neuroblastoma cells, and with c-MYC in pancreatic cancer cells (Liu et al., 2013). The activity of SIRT2 enhances N-MYC and c-MYC protein stability, promoting cancer cell proliferation.

### Mitochondrial Sirtuins

SIRT3, SIRT4, and SIRT5 are known as mitochondrial SIRTs (mtSIRTs) and are involved in the regulation of many biological and physiological processes such as cell cycle, cell viability, stress response, energy homeostasis, and metabolism (Lombard et al., 2011). mtSIRTs are critical regulators of metabolic functions, and act as checkpoints for mitochondrial membrane integrity by inhibiting the translocation of the pro-apoptotic protein BAX. mtSIRTs can also regulate cell survival and death by controlling metabolic state of cells. Since tumorigenesis involves several alterations in cellular and mitochondrial energy metabolism (Parihar et al., 2015), the role of mtSIRTs in tumor initiation is crucial. Several studies reported that alterations of mitochondrial metabolism lead to an increase in reactive species of oxygen (ROS) production, a key event in both aging and cancer. At low levels, ROS act as second messengers, stimulating cell proliferation and aggressive phenotype, preventing apoptosis, and promoting tumorigenesis (Murphy, 2013; Sullivan and Chandel, 2014).

#### SIRT3

SIRT3 is the best-characterized mtSIRT. It is principally located in mitochondria, where under stress conditions it translocates into the mitochondrial matrix, and after a proteolytic process is activated (Onyango et al., 2002; Table 1). Although some studies describe its possible localization in the nucleus, its function in this compartment is not yet clarified. Nuclear SIRT3 is reported to be a possible regulator of gene expression after cellular stress (Iwahara et al., 2012). SIRT3 controls the acetylation status of different proteins regulating mitochondrial metabolism, oxidative stress, and ROS production, thereby preventing apoptosis, growth arrest, and senescence, and promoting cancer cell proliferation (Park et al., 2011). Cell stress causes damage to mitochondria, leading to a reduction in SIRT3 activity, correlating with a decrease in deacetylation and NAD/NADH ratio and growth arrest (Hershberger et al., 2017). Several studies describe a crucial role for SIRT3 in cancer development and progression. In different types of cancers including colon, gastric, renal, oral squamous, and esophageal cancer and melanoma, SIRT3 expression is upregulated (Finley and Haigis, 2012; Liu et al., 2014), determining an alteration of many important biological processes and correlating with high tumor grade, positive lymph node status, and poor prognosis. In bladder carcinoma, SIRT3 promotes proliferation by abrogating the anti-proliferative activity of P53 in mitochondria (Li et al., 2010). In cervical cancer, its interaction with KU70 alters the DNA repair pathway. SIRT3 was also reported to have a dual function in tumorigenesis, due to its deacetylation activity of mitochondrial proteins such as SOD2, IDH2, and FOXO3a, and inhibition of mitochondrial ROS production and cancer cell proliferation (Torrens-Mas et al., 2017). The treatment of two different leukemia cell lines with a natural flavonoid, kaempferol, increases the expression and mitochondrial localization of SIRT3, resulting in AKT pathway inactivation as well as cytochrome c release and apoptotic cell activation (Marfe et al., 2009). In human breast and gastric cancer, downregulation of SIRT3 correlates with upregulation of HIF-1α and cell proliferation arrest (Finley et al., 2011; Yang et al., 2014).

#### SIRT4

SIRT4 is principally involved in genome stability and metabolism. Unlike other SIRTs, SIRT4 does not have nicotinamide adenine dinucleotide-dependent deacetylase activity, but does display ADP-ribosylase activity (Table 1). SIRT4 also negatively regulates mitochondrial glutamine metabolism by inhibiting glutamate dehydrogenase activity (Haigis et al., 2006). SIRT4-associated function on glutamine metabolism contributes to the control of cell cycle progression and proliferation, and is important for genomic integrity in response to DNA damage (Jeong et al., 2013). SIRT4 exerts tumor suppressor activity, arresting cell cycle and inhibiting invasion, proliferation, and cell migration. Although few studies have analyzed the role of SIRT4 in tumorigenesis to date, low expression of SIRT4 is reported in many cancers such as bladder, breast, colon, stomach, ovarian, and thyroid cancer, and correlates with a worse prognosis (Jeong et al., 2013; Huang and Zhu, 2018). SIRT4 loss leads to both increased glutamine-dependent proliferation and stress-induced genomic instability, resulting in tumorigenic phenotype. In colorectal cancer, SIRT4 overexpression was shown to suppress malignancy by blocking cancer cell proliferation via association with E-cadherin (Miyo et al., 2015). In breast cancer, loss of SIRT4 correlates with short time to metastasis development (Shi et al., 2016). In Burkitt lymphoma, SIRT4 is able to block MYC-driven lymphomagenesis by inhibiting mitochondrial glutamine metabolism (Jeong et al., 2014).

#### SIRT5

To date, only one physiological substrate for SIRT5 has been identified, carbamoyl-phosphate synthetase 1, an enzyme that plays an important role in urea cycle (Kumar and Lombard, 2015). Other enzymatic activities for SIRT5 such as desuccinylation, glutarylation, and demalonylation were recently characterized (Du et al., 2011; Table 1). Although the role of SIRT5 in tumorigenesis is not yet well characterized, it is likely related to its desuccinylation activity and to induction of the antioxidant enzyme SOD1 involved in ROS production. SIRT5 is overexpressed in human non-small cell lung cancer, and its role was recently investigated in colorectal cancer (Lu et al., 2014). High expression of SIRT5 is a predictor of poor survival, facilitating cancer cell growth and drug resistance. Accordingly, SIRT5 knock-down makes lung cancer cells more sensitive to chemotherapy (Wang et al., 2018).

## Sirtuin Modulators in Cancer

A growing body of evidence in recent years suggests that SIRT deregulation is involved in human carcinogenesis, placing these enzymes at the center of extensive pharmacological investigations such as drug target studies (Blum et al., 2011). SIRT activity can be modulated by several molecules, and high-throughput and *in silico* screenings have identified a number of small SIRT inhibitor (SIRTi) and activator compounds. In terms of their potential application in cancer treatment, the most promising data have been generated from the use of SIRTi rather than activators.

### Sirtuin Inhibitors

Besides nicotinamide, a few SIRTi displaying therapeutic potential in several human diseases have been developed (Carafa et al., 2012, 2018). To date, little is known about their specific anticancer action. SIRTi are grouped according to their pharmacophore type: β-napthol, indole, urea and thiourea derivatives, and miscellaneous.

Sirtinol is a β-napthol derivative identified by a high-throughput phenotypic screen in cells (Grozinger et al., 2001) which exerts anticancer activity via p53 acetylation (Vaziri et al., 2001; Peck et al., 2010) and senescence-like growth arrest (Ota et al., 2006). Sirtinol treatment is also able to increase cancer cell sensitivity to camptothecin and cisplatin (Kojima et al., 2008; Jin et al., 2010). Splitomicin is another β-napthol derivative identified by a cell-based screen for Sir2p inhibitors (Bedalov et al., 2001), but has a weak action on human SIRTs. In contrast, splitomicin analogs have shown activity against SIRT1 and SIRT2 as well as antiproliferative properties in cancer cells (Neugebauer et al., 2008; Freitag et al., 2011). Cambinol is the most stable and effective β-napthol derivative. It inhibits SIRT1, SIRT2, and, weakly, SIRT5 (Heltweg et al., 2006). Cambinol displays antitumor activity *in vitro* and in a Burkitt lymphoma mouse xenograft model by hyperacetylating tubulin, p53 and BCL6 (Heltweg et al., 2006). Salermide is able to induce a strong anticancer effect by reactivating proapoptotic genes through SIRT1-mediated K16H4 deacetylation (Lara et al., 2009). Its derivatives show broad-spectrum anticancer properties (Rotili et al., 2012). EX-527, the most well-described indole derivative, was identified by high-throughput screening against human SIRT1 (Napper et al., 2005). This inhibitor acts after the release of nicotinamide from the SIRT1 enzyme and prevents the release of deacetylated peptide and *O*-acetyl-ADP-ribose, products of the SIRT reaction. Thanks to its chemical properties (low molecular weight, cell permeability, oral bioavailability, and metabolic stability), EX-527 has been used to study the biology of SIRT1 and to explore therapeutic applications for SIRT1 inhibitors. This SIRT1-selective inhibitor showed a strong antiproliferative effect in pancreatic cancer cells and enhanced sensitivity of cancer cells to gemcitabine treatment through increased apoptosis (Zhang et al., 2014; Oon et al., 2015). Suramin is a polyanionic urea derivative used for treatment of trypanosomiasis (Voogd et al., 1993) and is also a potent anticancer agent that acts by inhibiting cell proliferation and angiogenesis (Stein et al., 1989; Li et al., 2015). However, its neurologic toxicity has limited its use in the clinic. Suramin analogs have been synthesized, and show a more potent antiangiogenic action (Meyers et al., 2000). In addition, in non-toxic doses, suramin potentiates the *in vitro* and *in vivo* activity of chemotherapeutics (Song et al., 2000; Zhang et al., 2001; Villalona-Calero et al., 2008), accounting for its use in several completed phase 1/2 clinical trials involving patients with solid tumors. Tenovins, SIRT1/2 inhibitors, are thiourea derivatives identified as a class of small molecules able to activate p53 and decrease tumor growth (Lain et al., 2008). Their water soluble analog is Tenovin 6, a promising agent for treating uveal melanoma (Dai et al., 2016). MC2494, a new pan-SIRTi, was recently found to display promising anticancer activity via acetylated RIP1/caspase-8-mediated apoptosis. MC2494 displays tumor-selective potential *in vitro*, in leukemic blasts *ex vivo*, and *in vivo* in both xenograft and allograft cancer models (Carafa et al., 2018).

### Sirtuin Activators

High-throughput screening identified small molecule SIRT-activating compounds (STACs). Resveratrol and other polyphenols were the first STACS to be discovered (Howitz et al., 2003), followed by an increasing number of molecules of doubtful activity (Baur and Sinclair, 2006). Other phenolic STACs include butein, piceatannol, isoliquiritigenin, fisetin, and quercetin (Link et al., 2010). Although several efforts have focused on better understanding the effects of resveratrol in cancer (Baur and Sinclair, 2006), many aspects remain unclear and controversial (Hubbard and Sinclair, 2014). In 1997, a report described the ability of resveratrol to inhibit development of preneoplastic lesions in carcinogen-treated mouse mammary glands in culture and tumorigenesis in a mouse skin cancer model (Jang et al., 1997). Subsequent studies showed that the anticancer activity of resveratrol was mediated at least in part by SIRT1 (Boily et al., 2009). Resveratrol reduces the proliferation of different cancers (Aggarwal et al., 2004; Firestein et al., 2008). Conversely, resveratrol fails to do so in breast cancer (Bove et al., 2002) and in multiple myeloma (Popat et al., 2013). This contrasting anticancer effect of resveratrol may be due to different doses used and its bioavailability in different tissues (Baur and Sinclair, 2006) as well as to its pharmacokinetic properties (Boocock et al., 2007). Recently, major efforts have focused on non-polyphenolic SIRT1 activators, including new second-generation (SRT2183, SRT1460, and SRT1720) and third-generation (STAC-5, STAC-9, and STAC-10) small molecules. Again, their anticancer effects remain elusive. SRT1720 was reported to induce cell death in breast cancer (Lahusen and Deng, 2015) and in multiple myeloma cells (Chauhan et al., 2011), but also to promote tumor cell migration and lung metastasis of breast cancer (Suzuki et al., 2012).

## Conclusion

Sirtuins are known to be involved in several biological processes, and play a critical role in contributing to the hallmarks of cancer. The dual role of SIRTs in tumor initiation, promotion, and progression may depend on their tissue- and cancer-specific expression as well as experimental conditions. A better characterization of each SIRT will enhance our understanding of their specific role in altered cancer signaling pathways. Targeting these epigenetic modulators may sensitize malignant cells to anticancer treatment, increasing the cytotoxic effect of chemotherapeutics and reducing tumor cell proliferation. Depending on their specific function as tumor promoters or tumor suppressors, a greater insight into SIRT modulators may open the way toward tailored medicine either inhibiting or activating SIRT action in a tumor-specific manner. The effects of SIRTs on cell energy metabolism that contribute to determining cellular microenvironment both in normal and pathological conditions add another layer of complexity to our understanding of their dual function. Further studies are required to shed light on this intricate role of SIRTs in different cancers and cell types in order to clarify in which conditions a specific SIRT functions as a tumor promoter or suppressor. Although several SIRT modulators, mainly inhibitors, were developed and tested *in vitro* (Carafa et al., 2012), few have been investigated *in vivo*. To date, only one phase 2/3 clinical trial (recruiting) is currently studying the combinatorial effect of the SIRTi nicotinamide in modulating SIRT activity in non-small cell lung carcinoma (NCT02416739).

The discovery of novel SIRT-selective modulators will help elucidate the role of individual SIRTs in cancer development and unveil other downstream target genes in cancers involving SIRT regulation.

Given the opposing role of SIRTs in cancer, it is crucial that future investigations focus on the undesired potential of SIRTs (and their modulators) to act against one cancer while promoting the genesis of other tumor types. Advanced technologies, such as the discovery of selective biomarkers for individual cancer types, the development of effective precision cancer therapies, and targeted delivery systems, will help address this concern and drive future clinical practice toward non-invasive, personalized, and controllable therapies.

## Author Contributions

VC, LA, and AN substantially contributed to the redaction of the manuscript and gave the final approval of the manuscript.

## Conflict of Interest Statement

The authors declare that the research was conducted in the absence of any commercial or financial relationships that could be construed as a potential conflict of interest.
